# Selective activation of PPARα maintains thermogenic capacity of beige adipocytes

**DOI:** 10.1016/j.isci.2023.107143

**Published:** 2023-06-19

**Authors:** Gentaro Egusa, Haruya Ohno, Gaku Nagano, Junji Sagawa, Hiroko Shinjo, Yutaro Yamamoto, Natsumi Himeno, Yoshimi Morita, Akinori Kanai, Ryuta Baba, Kazuhiro Kobuke, Kenji Oki, Masayasu Yoneda, Noboru Hattori

**Affiliations:** 1 Department of Molecular and Internal Medicine, Graduate School of Biomedical & Health Sciences, Hiroshima University, Hiroshima, Japan; 2 Department of Preventive Medicine for Diabetes and Lifestyle-related Diseases, Graduate School of Biomedical and Health Sciences, Hiroshima University, Hiroshima, Japan; 3 Department of Computational Biology and Medical Sciences, Graduate School of Frontier Sciences, The University of Tokyo, Tokyo, Japan

**Keywords:** Molecular biology, Cell biology, Animal Physiology

## Abstract

Beige adipocytes are inducible thermogenic adipocytes used for anti-obesity treatment. Beige adipocytes rapidly lose their thermogenic capacity once external cues are removed. However, long-term administration of stimulants, such as PPARγ and β-adrenergic receptor agonists, is unsuitable due to various side effects. Here, we reported that PPARα pharmacological activation was the preferred target for maintaining induced beige adipocytes. Pemafibrate used in clinical practice for dyslipidemia was developed as a selective PPARα modulator (SPPARMα). Pemafibrate administration regulated the thermogenic capacity of induced beige adipocytes, repressed body weight gain, and ameliorated impaired glucose tolerance in diet-induced obese mouse models. The transcriptome analysis revealed that the E-twenty-six transcription factor ELK1 acted as a cofactor of PPARα. ELK1 was mobilized to the *Ucp1* transcription regulatory region with PPARα and modulated its expression by pemafibrate. These results suggest that selective activation of PPARα by pemafibrate is advantageous to maintain the function of beige adipocytes.

## Highlights

• PPARα activation by pemafibrate maintains beige adipocytes’ thermogenic function
• Pemafibrate improved the metabolic phenotype in DIO mice with beige fat
• ELK1 is an essential cofactor of PPARα in pemafibrate-regulated gene expression

## Graphical abstract

**Figure:**
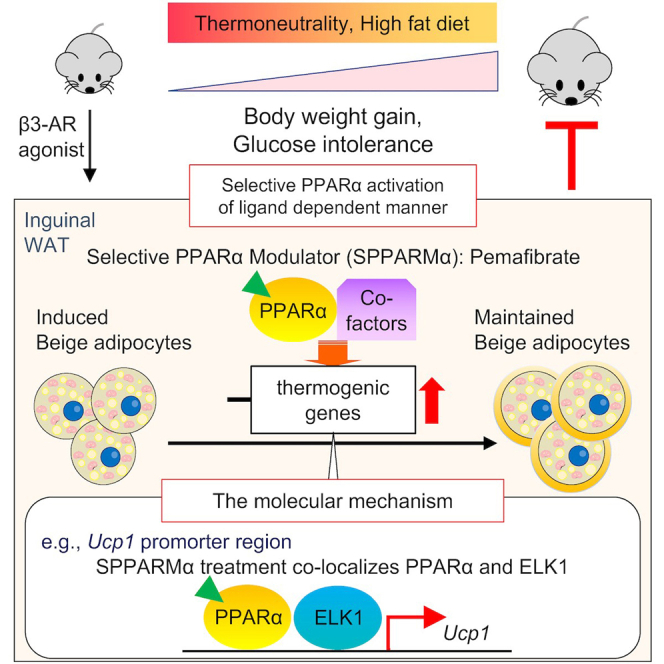

## Introduction

Thermogenic adipocytes, including brown and beige, have emerged as targets for treating metabolic disorders. These thermogenic adipocytes have abundant mitochondria and a unique protein, uncoupling protein-1 (UCP1), which produces heat by uncoupling the electron transport chain from ATP synthesis.^1^^,^^2^ In particular, beige (or brite) adipocytes can be induced in white adipose tissue (WAT) by various external cues such as chronic cold exposure or long-term administration of peroxisome proliferator-activated receptor γ (PPARγ) agonists in rodent models. In humans, 2-deoxy-2-(^18^F)fluoro-D-glucose ([^18^F]FDG) PET scanning has revealed that metabolically active brown adipose tissue (BAT) exists even in adults^3^^,^^4^^,^^5^^,^^6^^,^^7^ and possesses murine beige-like molecular characteristics in the supraclavicular and neck regions.^8^^,^^9^^,^^10^ Therefore, understanding the mechanism of beige adipocyte development might be linked to its practical application in human BAT activation.

The development of brown and beige adipocytes is regulated by various transcriptional regulatory factors, such as PR (PRD1–BF1–RIZ1 homologous) domain-containing 16 (PRDM16),^11^^,^^12^ early B cell factor 2 (EBF2),^13^ or nuclear factor I-A (NF1A).^14^ In addition, euchromatic histone lysine methyltransferase 1 (EHMT1) regulates the development of brown and beige adipocytes by interacting with the PRDM16 transcriptional complex and epigenetically modulating the reduced expression of myogenic and white adipogenic genes.^15^^,^^16^^,^^17^ Although the thermogenic capacity of induced beige adipocytes is almost the same as that of brown adipocytes, beige adipocytes lose their thermogenic function and de-differentiate into white adipocytes rapidly once developmental cues have been withdrawn.^18^ β3-Adrenocepttor agonists or certain PPARγ agonists, such as rosiglitazone, can activate thermogenic programs. While these agonists strongly induce beige adipogenesis in rodent models, their long-term use in humans leads to adverse side effects, such as elevation of blood pressure or tachycardia by β3-adrenoceptor agonists and edema or heart failure by rosiglitazone.^19^^,^^20^ Therefore, it is necessary to identify valuable targets for beige biogenesis and develop drugs that can be used for a long time without unfavorable side effects.

Peroxisome proliferator-activated receptor α (PPARα) is a nuclear receptor protein that regulates lipid metabolism by controlling fatty acid oxidation and is expressed ubiquitously in the liver and BAT.^21^^,^^22^^,^^23^ Recently, the pharmacological modulation of PPARα to activate thermogenesis in thermogenic adipocytes has attracted attention. PPARα activation leads *Ucp1* gene expression in brown adipocytes.^24^ PPARα agonists, such as clofibrate, fenofibrate, and bezafibrate, clinically used for hypercholesterolemia or mixed dyslipidemia, induce and activate thermogenic adipocytes.^24^^,^^25^^,^^26^ Other PPARα agonists, such as GW9578, rescue the loss of beige adipocytes in lysine-specific demethylase 1 (LSD1)-knockout (KO) mice.^27^ Pemafibrate, a newly developed selective PPARα modulator (SPPARMα), has been approved in Japan since 2017 for the treatment of dyslipidemia. Pemafibrate is more selective to PPARα target genes and has fewer side effects than preexisting PPARα agonists.^28^^,^^29^

In this perspective, we describe the involvement of PPARα in the maintenance of beige adipocytes. Then, we show that pemafibrate is useful for maintaining the thermogenic capacity of beige adipocytes and ameliorates the metabolic phenotype of diet-induced obese (DIO) mice. Finally, we investigate the molecular mechanism of pemafibrate-driven thermogenic induction and identify a transcription factor that regulates *Ucp1* expression associated with PPARα.

## Results

### Beige adipocytes rapidly lost their thermogenic characteristics after the withdrawal of external cues, along with decreased expression of *Ppara*

**Figure 1:**
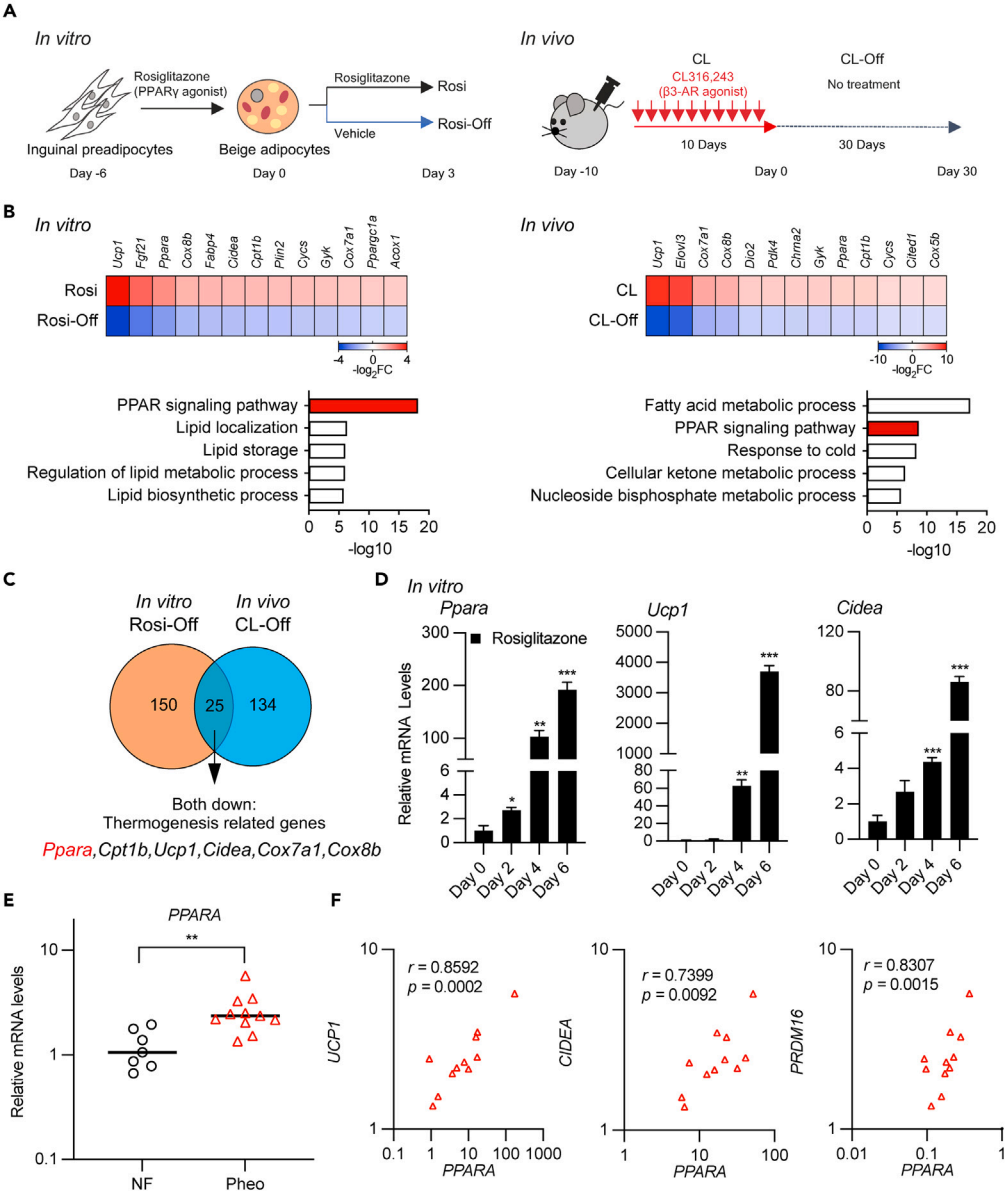
Beige adipocytes rapidly lose their thermogenic characteristics after the withdrawal of external cues along with decreased expression of Ppara (A) Schematic illustration of experiments. Induced beige adipocytes were treated with rosiglitazone (1 μM) *in vitro* and β3 agonist (1 mg kg^−1^) *in vivo*. After the introduction of beige adipocytes, the global RNA levels in beige adipocytes and inguinal beige fats between the induced models and withdrawn models (n = 3) were compared by RNA-sequencing. (B) Heatmap of representative thermogenic genes downregulated by the withdrawal of external stimuli. The log_2_-fold changes are shown in color scale. Pathway enrichment analysis of downregulated genes using Metascape. (C) Venn diagram of downregulated thermogenic genes. (D) Relative mRNA levels of *Pparα* and thermogenic genes during beige adipocyte differentiation by treatment with rosiglitazone (1 μM) *in vitro* (n = 4; mean ± SEM). (E) Relative mRNA levels of human *PPARA* in perirenal fat samples of human patients with pheochromocytoma or non-functional adrenal tumors (mean ± SEM; n = 7 independent samples for non-functional adrenal tumors and n = 11 independent samples for pheochromocytoma). (F) Correlation of the thermogenic related genes and *PPARA* expression in perirenal fat samples with pheochromocytoma.

Some synthetic compounds induce beige adipogenesis. For example, rosiglitazone strongly induces beige biogenesis by stabilizing the PRDM16 protein^30^ or continuous treatment with CL316,243, a β3-adrenergic agonist, leads to the development of thermogenic adipocytes in WAT and suppresses diet-induced obesity in mice.^31^ Using these beige-inducible compounds, we explored a key transcription factor for maintaining thermogenic capacity in beige adipocytes by comparing the characteristics of rosiglitazone-induced beige adipocytes *in vitro* and CL316,243-induced beige adipocytes *in vivo* after withdrawal of these stimuli (Figure 1A). Treatment of immortalized inguinal adipocytes with rosiglitazone for 6 days and injecting CL316,243 into mice for 10 days considerably increased the expression of thermogenic genes, such as *Ucp1*, *Cidea,* or *Ppara*. However, after the removal of the stimuli, these genes rapidly decreased in a time-dependent manner, as evaluated by quantitative PCR analyses (Figures S1A and S1B). Further, we obtained transcriptome data from the RNA-sequencing of the two groups. In the rosiglitazone-treated group, the expression levels of 150 genes, including *Ucp1*, *Cidea,* or *Ppara*, were remarkably downregulated to less than half after the removal of rosiglitazone. Similarly, in the CL316,243-treated mice, the expression levels of 134 genes, including some thermogenic genes, were remarkably downregulated by less than a quarter after the removal of CL316,243 stimulus (Figures 1B and S1C, upper panel). From these *in vitro* and *in vivo* data, pathway analyses revealed that PPAR signaling pathways were commonly downregulated after the removal of each stimulus (Figure 1B, lower panel). In addition, *Ppara* was the most downregulated transcription factor *in vitro* and was commonly reduced both *in vitro* and *in vivo* among the 25 genes (Figures 1C and S1D). PPARα is a major regulator of lipid metabolism in the liver. In adipose tissue, PPARα is expressed at relatively high levels in BAT than in WAT, and *Ucp1* is one of the PPARα target genes.^24^^,^^32^ Based on these results, we focused on PPARα involvement in transcriptional regulation to maintain thermogenic function in beige adipocytes. In the rosiglitazone-treated beige adipocyte differentiation process, *Ppara* significantly increased together with *Ucp1* and *Cidea*, according to the differentiation of beige adipocytes (Figure 1D). We previously reported that perirenal adipose tissue in humans with pheochromocytoma has the molecular characteristics of induced thermogenic adipocytes.^15^ Patients with pheochromocytoma, secreting catecholamines continuously, can be considered a chronic adrenergic-stimulating human model of adipocytes. *PPARA* expression levels in perirenal adipose tissue were significantly higher in the pheochromocytoma group than in the non-functional adenoma group (Figure 1E). Furthermore, human *PPARA* was significantly correlated with thermogenic genes, such as *UCP1*, *CIDEA,* and *PRDM16,* suggesting that PPARA may play an important role in the development of thermogenic adipocytes, even in human adults (Figure 1F).

### Pemafibrate maintained the thermogenic capacity of the induced beige adipocytes in a ligand-dependent manner

**Figure 2:**
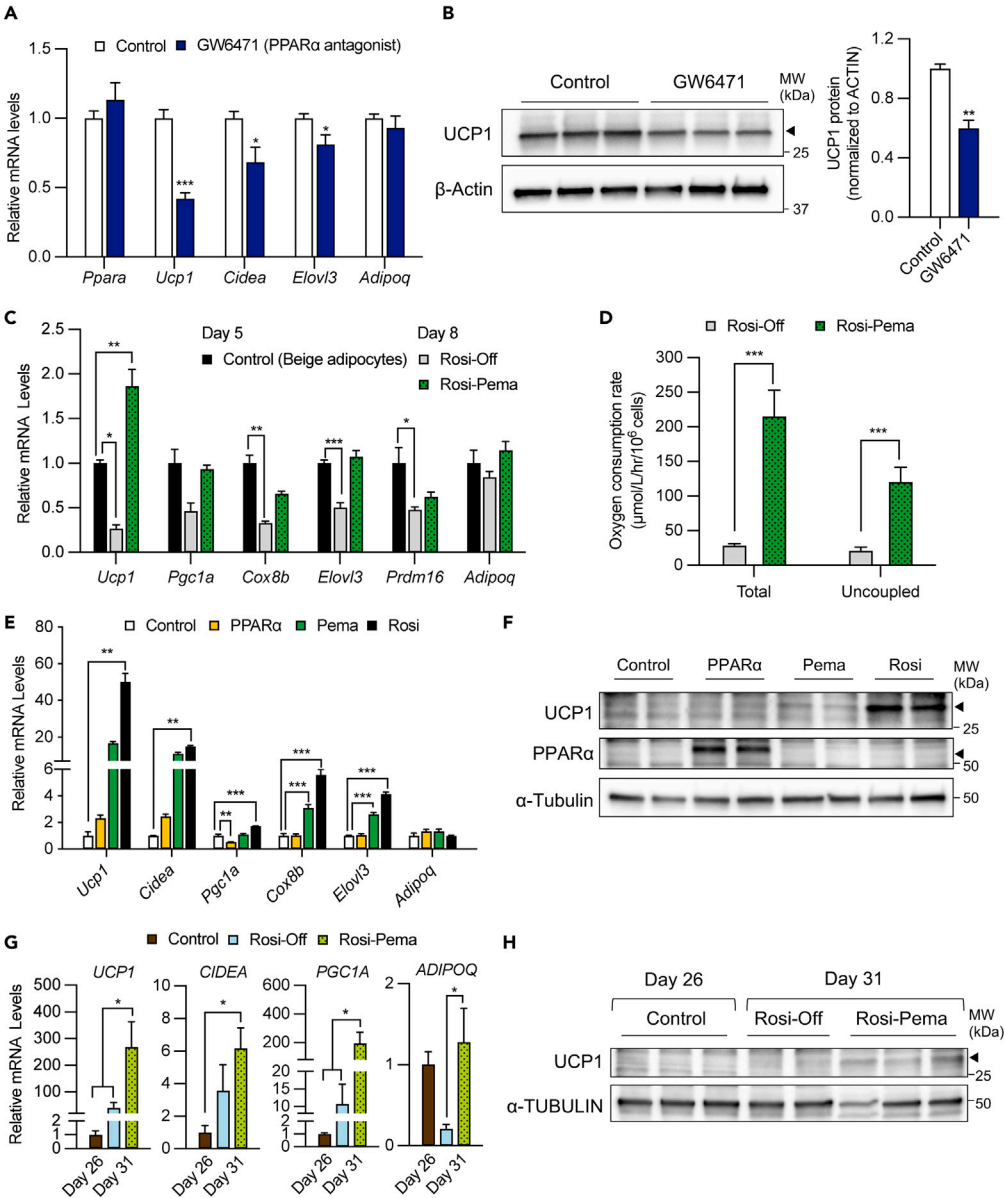
Pemafibrate maintained the thermogenic capacity of the induced beige adipocytes in a ligand-dependent manner (A) Relative mRNA levels of thermogenic genes in differentiated adipocytes with or without GW6471 (n = 4; mean ± SEM). (B) Western blot analysis (left panel) and quantification of UCP1 protein levels in differentiated adipocytes (right panel) (n = 4; mean ± SEM). The NIH Image J program was used to quantify the intensity of signals. (C) Relative mRNA levels of thermogenic genes vs. those of genes in the Rosi group on day 5 (n = 3. mean ± SEM). (D) Oxygen consumption rate in beige adipocytes switched to a vehicle or pemafibrate (10 μM) for 4 days (n = 4. mean ± SD). (E) Relative mRNA levels of thermogenic genes in differentiated adipocytes on day 6 vs. control. (n = 4; mean ± SEM). (F) Western blot analysis of UCP1 and PPARα protein levels. α-Tubulin was used as a loading control on day 6. (G) Relative mRNA levels of thermogenic genes in differentiated human adipocytes in the control group on day 26 vs. the Rosi-Off (vehicle) or Rosi-Pema (10 μM) groups on day 31 (n = 4; mean ± SEM). (H) Western blot analysis of UCP1 and α-Tubulin protein levels in the control, Rosi-Off, and Rosi-Pema groups. α-Tubulin was used as a loading control.

To investigate whether PPARα contributes to the maintenance of the thermogenic ability in beige adipocytes, we treated mature beige adipocytes with GW6471, a PPARα antagonist that suppresses the transcriptional activity of PPARα by recruiting co-repressors (Figure S2A).^33^ In the GW6471-treated group, the expression of thermogenic genes, such as *Ucp1*, *Cidea*, and *Elovl3,* and UCP1 protein decreased significantly (Figures 2A and 2B). Next, we investigated whether the pharmacological activation of PPARα enables the maintenance of the thermogenic function of beige adipocytes using PPARα agonists. First, beige adipocytes were treated with rosiglitazone for 5 days. Accordingly, rosiglitazone was withdrawn (Rosi-off) or switched to a PPARα agonist, such as bezafibrate (Rosi-Beza), GW9578 (Rosi-GW9578), or pemafibrate (Rosi-Pema) (Figure S2B). All PPARα agonists maintained *Ucp1* gene expression in beige adipocytes. However, pemafibrate treatment exhibited a considerably higher increase in *Ucp1* ratio compared to bezafibrate and GW9578 treatments (Figure S2C). Based on these results, we focused on the ability of pemafibrate to maintain thermogenic genes.

The expression of thermogenic genes significantly increased along with UCP1 protein expression in the Rosi-Pema group (Figures 2C and S2D). The oxygen consumption rate was also significantly increased in the Rosi-Pema group (Figure 2D). From these results, pemafibrate is suggested to enable the maintenance of the thermogenic capacity of beige adipocytes.

To examine the effect of pemafibrate on beige adipocyte induction, preadipocytes were treated with a vehicle (control), pemafibrate (Pema), and rosiglitazone (Rosi) in addition to a regular adipogenic cocktail (Figure S2E, left panel). We also overexpressed PPARα exogenously (PPARα) in preadipocytes at a significantly higher level than in the control, Pema, and Rosi groups (Figure S2E, right panel). Pemafibrate treatment increased the expression of thermogenic genes, including *Ucp1*, *Cidea*, *Cox8b*, and *Elovl3*, as well as the UCP1 protein, in differentiated adipocytes. Notably, the expression levels of these genes and protein were marked higher in the Rosi group than in Pema group. In the PPARα group, thermogenic genes and UCP1 protein were slightly increased compared to the control group, although they were not as high as those in the Pema and Rosi groups (Figures 2E and 2F).

These results suggest that the activation of thermogenic genes by pemafibrate treatment is not considerably greater than that by rosiglitazone treatment and that the induction of thermogenic genes is dependent on the activation of PPARα by its ligand, rather than the mere presence of the PPARα protein.

Additionally, we investigated whether pemafibrate maintains thermogenic genes in human brown adipocytes using the immortalized preadipocyte cell line hTERT A41hBAT-SVF.^34^ The preadipocytes were sufficiently differentiated using BMP7 (days 1–6) and rosiglitazone (days 7–20). Rosiglitazone was then withdrawn (Rosi-off) or switched to pemafibrate (Rosi-Pema) for additional 6 days and compared to those in the control group on day 26 (Figure S2F). Pemafibrate treatment significantly increased the expression of *Ucp1* mRNA and UCP1 protein, highlighting the effectiveness of pemafibrate in enhancing the thermogenic program of human brown adipocytes.

### Pemafibrate treatment suppressed glucose intolerance and weight gain in DIO mice while maintaining beige adipocytes

**Figure 3:**
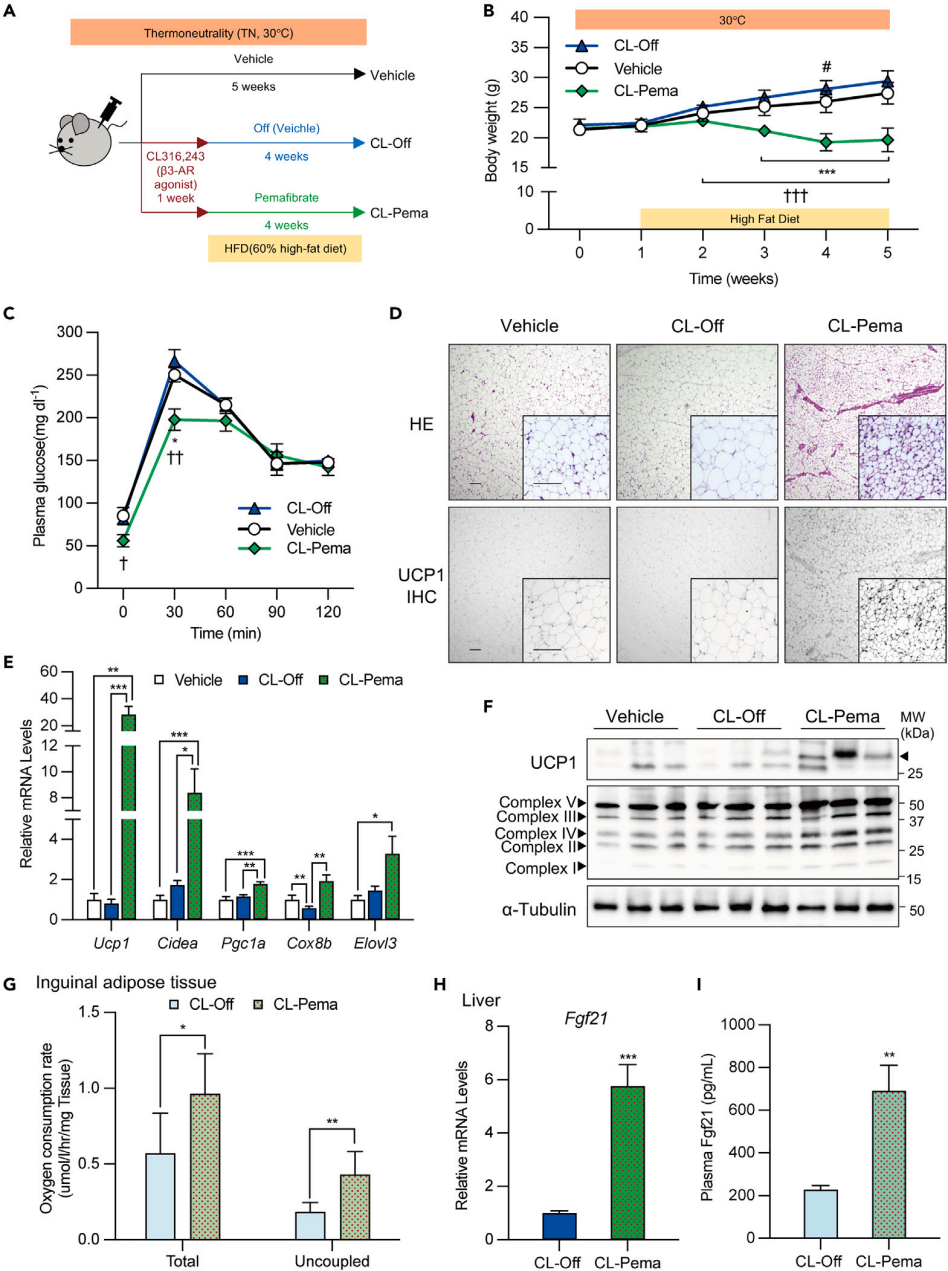
Pemafibrate treatment in DIO mice after the induction of beige fat in thermoneutral conditions (A) Schematic illustration of experiments. WT B6/J male mice were acclimated to thermoneutrality (TN, 30°C) for 1 week, treated with CL316,243 (1 mg kg^−1^) for 1 week, and switched to pemafibrate (Pema group; 1 mg kg^−1^) or a vehicle (Off group) for 4 weeks with high-fat diet. The group treated with only the vehicle under the same conditions during the experiment was used as the control (Vehicle). (B) Body weight change of each group of mice. Body weight was measured once a week (n = 8–10 per group; mean ± SD). (C) Plasma glucose levels following a glucose tolerance test (IPGTT, 1 g kg^−1^) in each group of mice on week 12 (n = 8–10 per group; mean ± SEM). (D) Hematoxylin & Eosin and UCP1 immunohistochemical staining in iWAT of control, Off, and Pema group mice. Scale bar, 100 μm. (E) Relative mRNA levels of thermogenic genes in iWAT (n = 7–10 per group; mean ± SEM). (F) Western blot analysis of UCP1 protein levels. (G) Oxygen consumption rates in *ex vivo* inguinal adipose tissue in CL-Off or CL-Pema (1.0 mg/kg) mice. (H) Relative *Fgf21* mRNA levels in the liver (n = 7–10 per group; mean ± SEM). (I) Plasma Fgf21 concentrations after vehicle or pemafibrate administration for 1 week in a beige adipocyte-induced mouse model (n = 4–5 per group; mean ± SEM). (B and C) Vehicle vs. CL-Pema (∗), CL-Off vs. CL-Pema (^†^), vehicle vs. CL-Off (^#^).

Next, we tried to elucidate whether pemafibrate could maintain the induced beige adipocytes in a mouse model. First, we investigated the thermogenic induction ability of pemafibrate (Pema) in inguinal adipose tissue compared to a vehicle (Vehicle), rosiglitazone (Rosi), or CL316,243 (CL) (Figure S3A). We found that the administration of rosiglitazone or CL316,243 for 10 days significantly induced thermogenic genes, including *Ucp1*, *Cidea*, or *Pgc1a*, but that pemafibrate treatment did not (Figure S3B). Hence, we aimed to examine the effect of pemafibrate after the induction of beige adipocytes by using CL316,243. After breeding mice at 30°C for a week, CL316,243 (1 mg kg^−1^) was administered for 1 week to induce beige adipocytes in the inguinal WAT. For the following 4 weeks, a vehicle (CL-Off) or pemafibrate (1 mg kg^−1^) (CL-Pema) was injected intraperitoneally. For control, a vehicle was administered intraperitoneally during all study periods (Vehicle). All mice were fed a high-fat diet (60% HFD) during the 4 weeks (Figure 3A). There was no significant difference in dietary intake between the groups (Figure S3C). HFD feeding induced body weight and glucose intolerance in the control and off groups; however, pemafibrate administration significantly repressed body weight gain and showed lower glucose levels, as estimated by the intraperitoneal glucose tolerance test (Figures 3B and 3C). To confirm whether beige adipocytes were maintained in subcutaneous WAT, inguinal WAT was collected. Histologically, adipocytes in the CL-Pema group had smaller lipid droplets and were UCP1-positive, as assessed by hematoxylin and eosin and immunohistochemical staining (Figure 3D). Thermogenic gene expression and protein levels of UCP1 and respiratory chain components were higher in the CL-Pema group than in the vehicle and off groups (Figures 3E and 3F). We also confirmed that oxygen consumption rates were increased in *ex vivo* inguinal adipose tissue in the CL-Pema group (Figure 3G). In the CL-Pema group, the expression of *Ucp1* in BAT increased slightly, and the expression of thermogenic genes in epididymal WAT increased significantly; however, UCP1 protein levels did not differ between BAT and epididymal WAT (Figures S3D and S3E). A previous study reported that pemafibrate administration in mice increased FGF21 production, leading to improvements in terms of body weight gain and glucose intolerance.^35^ Both liver *Fgf21* expression levels and plasma FGF21 concentrations demonstrated a significant increase following pemafibrate treatment (Figures 3H and 3I). These results suggest that induced beige adipocytes are sufficiently maintained with pemafibrate administration, which may contribute to the repression of glucose intolerance and inhibition of body weight gain in the DIO mouse model.

### ELK1 is an important cofactor of PPARα for pemafibrate-regulated thermogenic gene regulation

**Figure 4:**
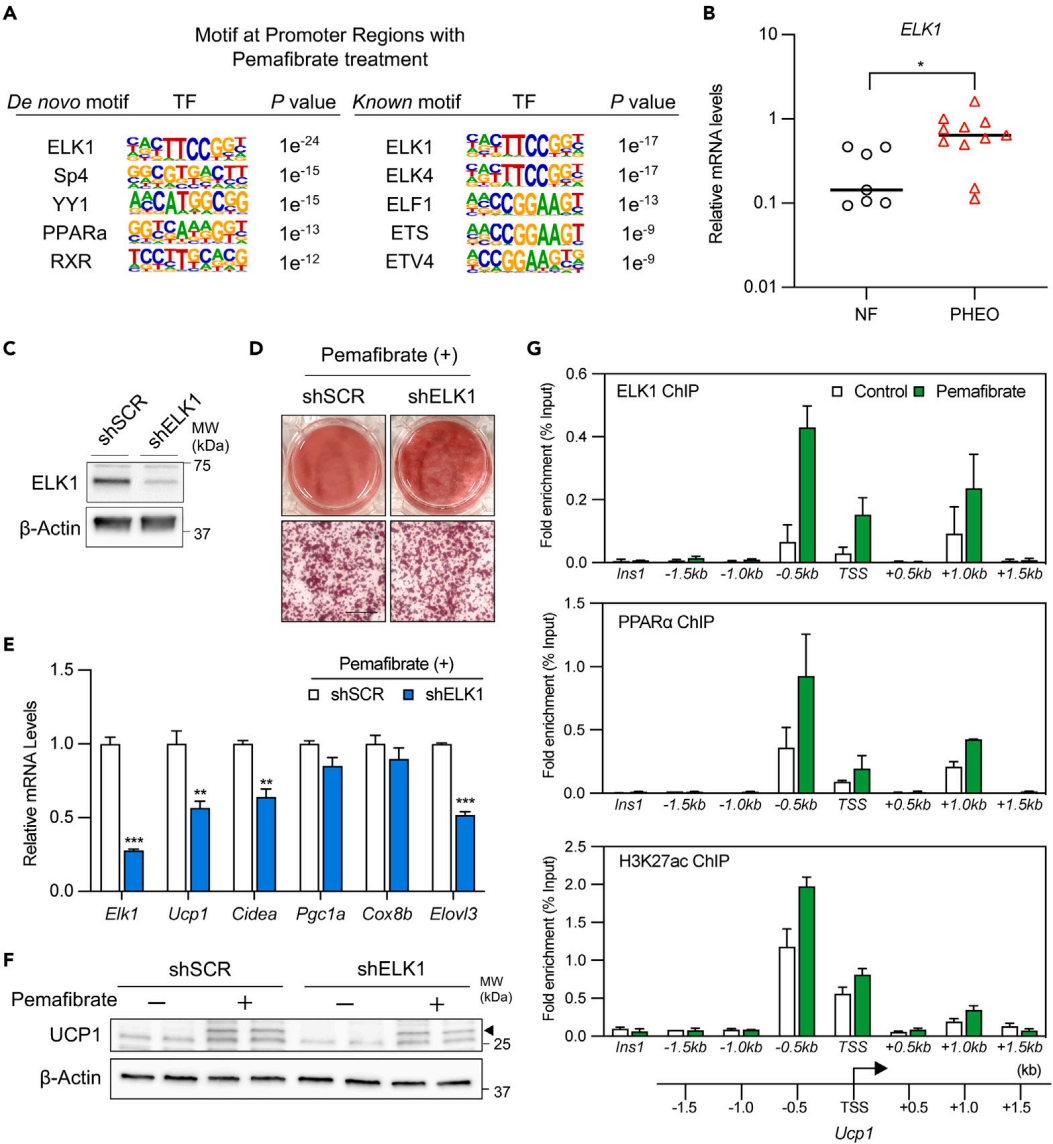
ELK1 is required for the regulation of thermogenic genes by pemafibrate (A) *De novo* and *Known* motif analysis of regions with upregulated gene levels by pemafibrate treatment. (B) Relative mRNA levels of human *ELK1* in perirenal fat samples of human patients with pheochromocytoma or non-functional adrenal tumors (n = 7 independent samples for non-functional adrenal tumors and n = 11 independent samples for pheochromocytoma; mean ± SEM). (C and D) Immunoblotting of immortalized inguinal adipocytes expressing shRNA for a scrambled control (SCR) or ELK1. β-Actin was used as a loading control. Oil Red O staining of differentiated adipocytes treated with pemafibrate expressing the indicated construct. Scale bar, 100 μm. (E) Thermogenic genes in differentiated adipocytes treated with pemafibrate (10 μM) expressing the indicated constructs (n = 4; mean ± SEM). (F) Western blot analysis of UCP1 protein levels. β-Actin was used as a loading control. (G) ChIP assay in pemafibrate differentiated adipocytes using specific antibodies against PPARα, ELK1, and H3K27ac. Fold enrichment at each Ucp1 promoter site assessed by qPCR compared with control (vehicle). *Ins1* was used as a non-specific binding site (n = 2; means ± SD).

PPARα agonists exert their transcription regulatory action via transcription cofactors, and each PPARα agonist has a unique agonist-specific type of cofactor mobilization.^36^ To elucidate the mechanism by which SPPARMα induces thermogenic programs, we investigated the binding state of transcription regulators on thermogenic genes induced by various PPAR agonists (Figure S4A). Rosiglitazone, pemafibrate, and GW9578, another selective PPARα agonist, significantly induced beige-enriched thermogenic and mitochondrial genes and repressed white adipocyte-specific markers, such as *Retn, Agt*, or *Lep* (Figures S4B and S4C). Motif analysis revealed the presence of ETS-related genes, such as *ELK1*, in the promoter regions of the Pema group (Figure 4A). Interestingly, the transcriptional factors present in the promoter region of the GW9578 group differed remarkably from those in the Pema group, and a multidimensional scaling (MDS) analysis revealed that the position of pemafibrate-regulated genes was distant from that of GW9578-regulated genes (Figures S4D and S4E). Motif analysis of rosiglitazone-regulated genes also revealed the presence of ETS-related genes in the promoter regions (Figure S4F). Because the effects of ELK1 on thermogenic adipocytes have not been reported, we examined the contribution of ELK1 to the pemafibrate-induced thermogenic gene programs. Gene expression of *Elk1* was also increased in drug-induced beige adipocytes *in vitro* and *in vivo* (Figures S3G and S3H). The gene expression of *ELK1* was also increased in the perirenal tissue of human pheochromocytoma and correlated with *PPARA* expression (Figures 4B and S4I). ELK1 reportedly forms a transcriptional complex together with MED23 and controls white fat differentiation; MED23 has been identified as a member of the PPARα transcription complex.^37^^,^^38^ In this study, we confirmed that PPARα and ELK1 form transcriptional complexes together with MED23 in the nucleus of beige adipocytes (Figure S4J). To determine whether ELK1 is required for the expression of thermogenic genes, we knocked down ELK1 using lentiviral shRNA in preadipocytes and differentiated them with pemafibrate treatment (Figure 4C). Oil Red O staining showed that adipogenesis was not altered with ELK1 depletion (Figure 4D); however, the expression of thermogenic genes and UCP1 protein were significantly reduced in ELK1-deficient cells (Figures 4E–4F), indicating that ELK1 is required for thermogenic gene regulation by pemafibrate treatment. In contrast, *ELK1* knockdown did not affect either the induction of thermogenic genes by PPARγ agonists or the activation of PKA by forskolin, indicating that ELK1 is specifically required for pemafibrate-caused effects on thermogenic gene induction (Figure S4K). When examining the effects of pemafibrate on ELK1 expression in differentiated adipocytes, pemafibrate slightly affected the ELK1 gene expression or protein levels (Figures S4L and S4M). Finally, to clarify the transcriptional regulation of *Ucp1*, a representative thermogenic gene, by PPARα and ELK1 with pemafibrate, we performed ChIP-qPCR analyses with antibodies against ELK1, PPARα, and H3K27ac, an active histone marker. Interestingly, ELK1 was enriched in the *Ucp1* transcription regulatory region with PPARα and H3K27Ac, and this enrichment was robustly increased by pemafibrate treatment, thereby suggesting that ELK1 and PPARα are co-localized in the regulatory region of *Ucp1.* PPARα agonism by pemafibrate treatment increases this co-localization and regulates *Ucp1* expression (Figure 4G). These results suggest that ELK1 is an important factor in the regulation of thermogenic genes transcriptionally controlled by pemafibrate treatment.

## Discussion

The present study demonstrated that the thermogenic capacity of pharmacologically induced beige adipocytes can be maintained via selective activation of PPARα by pemafibrate administration. The administration of PPARα agonist partially repressed the whitening of induced beige adipocytes in LSD1-deficient mice.^27^ PPARα is important for remodeling WAT by administering CL316,243.^39^ Some reports indicate that PPARα is not essential for thermogenesis in BAT,^23^^,^^40^ and the induction of beige adipocytes under cold stimuli.^41^ The action of PPARα differs in the developmental process of thermogenic adipocytes, depending on various stimuli (environmental factors, agonists, etc.).

The activation of PPARα by synthetic PPARα agonists results in a conformational change in the PPARα complex. This complex binds to the target gene locus with cofactors to regulate transcriptional activity.^29^ In the regulation of thermogenic genes, administration of WY-14643, a PPARα agonist, activates the transcriptional function of PPARα in the presence of PRDM16,^11^ and GW7647, another PPARα agonist, recruits the PPARα-PRDM16 complex to the PGC1α modulating locus.^42^ This suggests that the activation of PPARα by ligands is deeply involved in the regulation of thermogenic gene expression. In humans, pemafibrate has been suggested to be effective in the treatment of dyslipidemia and in reducing adverse events in liver and kidney function compared to fenofibrate.^43^^,^^44^ By using an FGF21 KO mouse model to examine the effects of pemafibrate on adipocytes, it was evident that administering pemafibrate to a DIO mouse model increases thermogenic gene expression in inguinal WAT via increased production of Fgf21 in the liver.^35^ FGF21 acts on paracrine/autocrine signaling and increases the expression of thermogenic genes in inguinal WAT.^45^ The improvement in weight gain and glucose intolerance may be due to the synergistic effect on metabolic organs, such as adipose tissue and the liver.

However, there are currently no reports examining the direct effects of pemafibrate on adipocytes in detail. This study revealed that pemafibrate treatment induced beige adipogenesis in a ligand-dependent manner. In addition, pemafibrate and GW9578 differed in the recruitment of transcription factors to the transcriptional regulatory regions based on motif analysis of RNA-seq. Furthermore, as one of the transcriptional cofactors with PPARα recruited by pemafibrate, ELK1 was identified. ELK1 is a ternary complex factor belonging to the ETS family of transcription factors. The ETS gene is conserved in various species and regulates cell differentiation, development, and proliferation^46^^,^^47^^,^^48^ ELK1 reportedly promotes the production of adipocytes via insulin-MAPK signaling in combination with MED23 (Sur2, DRIP130).^37^ In the nucleus of hepatocytes, MED23 binds to PPARα via the LXXLL motif.^38^ However, ELK1 has not been previously reported as a member of the PPARα transcription complex. The present study revealed that ELK1 forms a transcriptional complex with PPARα via MED23 in the nucleus of beige adipocytes and that pemafibrate treatment increases ELK1 enrichment in the transcriptional regulatory region of *Ucp1*. ELK1 may be a specific cofactor mobilized by pemafibrate administration, and the co-localization of PPARα and ELK1 may regulate the expression of *Ucp1*.

Clinically, pemafibrate can be safely used without rhabdomyolysis at various chronic kidney disease (CKD) stages. Several clinical studies have revealed that pemafibrate administration is not related to increased frequencies of side effects, such as tachycardia, edema, or body weight gain, which are commonly observed with β3-adrenergic agonists and PPARγ agonists.^49^^,^^50^

Furthermore, this study revealed that the pemafibrate treatment enhances thermogenic gene expression in human brown adipocytes. PPARα selective activation by pemafibrate might hold therapeutic potential for the treatment of obesity-related disorders.

In conclusion, we identified PPARα as a key factor for maintaining the thermogenic capacity of beige adipocytes. Further, we revealed that the transcription factor ELK1 co-localizes with PPARα and is involved in the transcriptional regulation of thermogenic genes. Pemafibrate is widely used in clinical practice, and our results shed light on its long-term usefulness in treating obesity-related disorders while avoiding side effects.

### Limitations of the study

The findings of this study suggest that pemafibrate is useful for maintaining mature beige adipocytes. However, the precise mechanism through which PPARα and ELK1 regulate the expression of thermogenic genes in response to pemafibrate remains unclear. Investigating the roles of PPARα and ELK1 in the mechanism of maintaining thermogenic capacity by pemafibrate was challenging in this study, particularly because of the technical difficulties in creating a loss-of-function model in mature beige adipocytes. In the future, studies using inducible ELK1 adipose tissue-specific knockout mice will demonstrate how ELK1 contributes to the mechanism by which pemafibrate induces the expression of thermogenic genes.

## STAR★Methods

### Key resources table

**Table:** 

REAGENT or RESOURCE	SOURCE	IDENTIFIER
**Antibodies**		

ELK1	Abcam	Cat# ab32106; RRID:AB_732141
PPARα	Gene Tex	Cat# GTX101098; RRID:AB_2037654
Sur-2	BD Pharmingen	Cat# 550429; RRID:AB_393678
DRIP130	Abcam	Cat# ab200351
Mouse (G3A1) mAb IgG1 Isotype Control	Cell signaling	Cat# 5415; RRID:AB_10829607
H3K27ac	Cell signaling	Cat# 81735
Anti-UCP1 antibody	Abcam	Cat# ab10983; RRID:AB_2241462
Anti-Total OXPHOS Rodent WB cocktail	Abcam	Cat# ab110413; RRID:AB_2629281
α-Tublin	Cell signaling	Cat# 2144; RRID:AB_2210548
β-Actin (13E5)	Cell signaling	Cat# 4970; RRID:AB_2223172
anti-rabbit IgG-Horseradish peroxidase (HRP)	GE Healthcare	Cat# NA934V
anti-mouse IgG-HRP	GE Healthcare	Cat# NA931V
Veriblot for IP Detection Reagent (HRP)	Abcam	Cat# ab131366; RRID:AB_2892718

**Biological samples**		

Fetal Bovine Serum	HyClone	Cat# SH30910.03

**Chemicals, peptides, and recombinant proteins**		

TRIzol Reagent	Thermo Fisher	Cat# 15596018
Complete, EDT-free Protease Inhibitor Cocktail	Roche	Cat# 04693132001
Halt Protease and Phosphatase Inhibitor	Thermo Fisher	Cat# 78442
TB Green Premix Ex Taq (Tli RNaseH Plus)	Takara bio	Cat# RR420A
PrimeScript RT Master Mix	Takara bio	Cat# RR036A
Lipofectamine RNAiMAX	Thermo Fisher	Cat# 13778-150
Opti-MEM	Life technologies	Cat# 31985-062
5×siRNA Buffer	Dharmacon	Cat# B-002000-UB-100
Insulin	SIGMA	Cat# I6634
3-Isobutymethylxanthine	SIGMA	Cat# I5879
Indomethacin	SIGMA	Cat# I7378
Dexamethasone	SIGMA	Cat# D1756
Triiodo-L-Thyronine (T3)	SIGMA	Cat# T-2877
Recombinant human BMP7 protein (Active)	Abcam	Cat# ab245953
Biotin	Wako	Cat# 029-08713
Sodium pyruvate solution 100 mM	SIGMA	Cat# S8636-100
Albumin, Bovine, Serum, Fatty acid free	Nacalai tesque	Cat# 08587-84
D-MEM (High glucose)	Wako	Cat# 043-30085
DMEM / F12+GlutaMAX-I	Life technologies	Cat# 10565-018
DMEM / F12 (1:1)	Life technologies	Cat# 11039-021
CL316,243	TOCRIS	Cat# 1499/10
Rosiglitazone	Tokyo Chemical Industry	Cat# R0106
Pemafibrate	Kowa, Tokyo, Japan	N/A
Bezafibrate	Wako	Cat# 022-16091
GW9578	SantaCruz	Cat# SC-221703
GW6471	Cayman	Cat# 11697
Recombinant Proteinase K Solution	Invitrogen	Cat# AM2546
RNaseA	NIPPON GENE	Cat# 313-01461
RNase,DNase free water	Invitrogen	Cat# 10977-015
Oligomysin	Cell signaling	Cat# 9996S
Antimycin	Sigma	Cat# A8674-25MG
Puromycin	Invivogen	Cat# ant-pr-1
G-418 Sulfate Solution	FUJIFILM	Cat# 077-06433
Penicillin-streptomycin solution	FUJIFILM	Cat# 168-23191
Ampicilin	Sigma	Cat# A5354
Oil red O stain stock solution	Muto Pure Chemicals Co	Cat# 4049-1
Collagenase D	Roche	Cat# 11088882001
Dispase II	Roche	Cat# 04942078001

**Critical commercial assays**		

Smartpool: ON-TARGET plus mouse Fgf21 siRNA	Dharmacon	Cat# L-063178-00-0005
Smartpool: ON-TARGET plus Non-targeting Pool siRNA	Dharmacon	Cat# D-001810-10-20
Mouse/Rat FGF-21 Quantikine ELISA Kit	R & D	Cat# MF2100
MinElute PCR Purification Kit	Qiagen	Cat# 28004

**Experimental models: Cell lines**		

Immortalized inguinal preadipocytes	This paper	N/A
hTERT A41hBAT-SVF	ATCC	Cat# CRL-3385

**Experimental models: Organisms/strains**		

Mouse:C57BL/6JJcl	CLEA Japan, Inc.	N/A

**Oligonucleotides**		

A full list of Primers see Table S1	This paper	N/A

**Recombinant DNA**		

pMEI5-neo-Flag-PPARα	This paper	N/A

**Deposited data**		

DNA-binding transcription factor activity	MGI	GO0003700
Gene Expression RNA-seq data	This paper	DRA014834, DRA014833 and DRA014840

**Software and algorithms**		

Metascape pathway analysis	Tripathi et al.^51^	N/A
GraphPad Prism 9	GraphPad Software	https://www.graphpad.com/scientific-software/prism/
Image J Java 1.6.0_24	NIH	https://imagej.nih.gov/ij/
HOMER		http://homer.ucsd.edu/homer/
EdgeR		https://bioconductor.org/packages/release/bioc/html/edgeR.htm

**Other**		

MF	Oriental Yeast Co.	N/A
High fat diet	Reserch diet	Cat# D12492

### Resource availability

#### Lead contact

Further information and request for resources and reagents should be directed to and would be fulfilled by the Lead Contact, Haruya Ohno (haruyan711@gmail.com).

#### Materials availability

Unique materials generated in this study are available upon completing the materials transfer agreement.

### Experimental model and study participant details

#### Animals

Eight-week-old male C57BL/6J mice were obtained from CLEA Japan, Inc. (Tokyo, Japan) (Figures 1, S3, and S4). Mice were intraperitoneally injected daily with 1 mg kg^−-1^ β3-AR agonist CL316,243 (TOCRIS#1499), 10 mg kg^-1^ PPARγ agonist rosiglitazone, or 1 mg kg^-1^ selective PPARα modulator pemafibrate for 10 days. Six-week-old male C57BL/6J wild-type (WT) mice were obtained from CLEA Japan, Inc. (Figure 3). Upon arrival, all animals were acclimated to thermoneutrality (TN, 30°C) for one week before randomization and fed an MF diet for two weeks at the start of interventions. To induce beige adipogenesis, CL316,243 was administered intraperitoneally to male mice at a dose of 1 mg kg^-1^ for 7 consecutive days. After 7 days, the treatment was switched to pemafibrate, provided by Kowa Company, Ltd. (Nagoya, Japan), at a dose of 1 mg kg^-1^ or vehicle for another 28 days with a HFD (Research Diet#D12492). For intraperitoneal glucose tolerance tests (IPGTT), mice were fasted for 6 h and then intraperitoneally administered glucose at a dose of 1 g kg^-1^ body weight after 4 weeks on HFD. The *in vivo* pemafibrate dosage used in this study was based on previous studies.^52^^,^^53^ *Ex vivo* experiments: Seven-week-old male C57BL/6J WT mice were obtained from CLEA Japan, Inc. (Figure 3G). Upon arrival, all animals were fed a normal chow diet and acclimated to TN (30°C) for one week before randomization. To induce beige adipogenesis, CL316,243 was intraperitoneally administered to male mice at a dose of 1 mg kg^-1^ for 7 consecutive days. After 7 days, the treatment was switched to pemafibrate (1 mg kg^-1^) or a vehicle for another 18 days.

Plasma FGF21 levels were analyzed using an ELISA kit (MF2100; R&D Systems, Minneapolis, MN, USA) (Figure 3I). All animal husbandry procedures and experiments complied with the University of Hiroshima’s Regulations for Animal Experiments and were approved by the Animal Experiment Committee of the University of Hiroshima on April 24, 2017.

#### Human subjects

Eleven subjects with adrenal pheochromocytoma and seven subjects with non-functioning adrenal tumors were enrolled in our previous study.^15^ The study was approved by the Hiroshima University Ethics Committee. All procedures were conducted according to the Declaration of Helsinki, and all patients provided written informed consent before participating in the study.

#### Cell cultures

Beige adipocyte cell lines were established by immortalizing stromal vascular fractions (SVFs) from inguinal WAT B6 WT male mice according to the cell immortalization protocol using the SV40 large T antigen.^54^ In brief, inguinal fat depots were digested in phosphate-buffered saline (PBS) containing collagenase D (1.5 U/mL) and dispase II (2.4 U/mL) supplemented with 10 mM CaCl_2_ at 37°C for 30 min. Primary cells were filtered through a 70 μm cell strainer and centrifuged at 700 × *g* to collect SVF. SV cell pellets were rinsed and plated on collagen-coated plates. Preadipocytes were immortalized by infection with the pMSCV-puro retroviral vector encoding the SV40T antigen and selection with puromycin (Invivogen# ant-pr-1, 2 mg/mL).

Immortalized mouse adipocyte cultures were prepared according to previously reported methods.^16^ Briefly, cells were cultured to confluence, and adipocyte differentiation was induced using a Dulbecco's Modified Eagle Medium (DMEM)/F-12 medium (with phenol red, Wako Pure Chemicals, Osaka, Japan) containing 10% fetal bovine serum (FBS), 1% penicillin–streptomycin, 5 μg/mL insulin, 1 nM T3, 0.25 mM IBMX, 0.125 mM indomethacin, and 2 μg/mL dexamethasone. Two days after induction, the cells were switched to a maintenance medium containing 10% FBS, 5 μg/mL insulin, and 1 nM T3 for an additional 5–6 days. To induce beige adipocyte differentiation, cells were differentiated in the presence of 1 μM rosiglitazone, as outlined in a previous study.^30^ The effects of PPARα transcriptional activity inhibition were determined using GW6471. The differentiated beige adipocytes were treated with 5 μM GW6471 to test its effect on thermogenic gene levels. The *in vitro* dosage of pemafibrate used in this study was based on a previous study.^55^ Pemafibrate (10 μM) was used to differentiate adipocytes, which was switched to rosiglitazone on days 5–6. GW9578 (1 μM) and bezafibrate (500 μM) were used to test its effect on thermogenic gene levels, which was switched to rosiglitazone on day 5. Mycoplasma infections were not tested in any of these experiments.

#### Immortalized human cell culture

Immortalized human progenitor cells (hTERT A41hBAT-SVF) were plated and grown in a DMEM/H medium supplemented with 10% FBS. For adipocyte differentiation, cells were grown for 6 days until reaching confluence; recombinant BMP7 (3.3 nM) was then added to undifferentiated cells in a DMEM-F12 medium containing insulin (0.5 μM), T3 (2 nM), and 2% FBS for 6 days. The cells were then exposed to an adipogenic induction mixture made up of isobutylmethylxanthine (0.5 mM), dexamethasone (0.1 μM), insulin (0.5 μM), T3 (2 nM), indomethacin (30 μM), rosiglitazone (1 μM), biotin (33 μM), and 2% FBS in a DMEM-F12 medium for another 19 days. After 20 days, the cells were switched to a DMEM-F12 medium containing dexamethasone (0.1 μM), insulin (0.5 μM), T3 (2 nM), and either a vehicle or pemafibrate (10 μM) for another 6 days.

### Method details

#### Histology

Samples were cut into 4 μm paraffin sections using a microtome (LS113; Yamato Kohki Industrial Co., Ltd., Saitama, Japan) and mounted on saline-coated glass slides (# 5166; Muto Pure Chemicals, Tokyo, Japan). Hematoxylin and eosin staining and immunohistochemistry were performed by Kyodo Byori Incorporation (Kobe, Japan) according to standard procedures. Immunohistochemistry was performed using anti-UCP1 (1:500, #ab10983; Abcam, Cambridge, UK) and anti-rabbit Histofine Simple Stain MAX PO (#424144; Nichirei Bioscience, Tokyo, Japan). All observations were performed using a KEYENCE BZ-9000 (Keyence, Tokyo, Japan).

#### Forward siRNA transfections

Using Dharmacon SmartPool On-Target PLUS RNA pools, immortalized inguinal preadipocytes were forward-transfected with siRNA as per the manufacturer’s protocol when the cells reached 80% confluence on collagen I-coated 12-well cell culture plates (4815-010; IWAKI) (Table S1). The siRNA and Lipofectamine RNAiMAX (13378-150; Invitrogen, Carlsbad, CA, USA) were diluted separately in Opti-MEM® I Reduced Serum Medium (31985-062; Life Technologies, Carlsbad, CA, USA) and mixed by pipetting. The siRNA-RNAiMAX mixture was incubated for 20 min at room temperature and was then added onto the adherent cells, and the cells were gently shaken. The final concentrations of Lipofectamine RNAiMAX and siRNA were 3 μL/mL and 20 nM, respectively. The preadipocytes were then harvested to reach confluence 1 day after transfection. The cells were then subjected to adipogenic conditions to promote mature adipocyte differentiation, with the addition of pemafibrate (10 μM) for 4 days. The cells were forward-transfected 3 days after the first transfection.

#### DNA constructs and virus production

The constructs were subcloned into a pMEI5-neo retroviral vector (Genscript, Piscataway, NJ, USA). For retrovirus production, Phoenix packaging cells were transfected at 70% confluence using the polyethylenimine "Max" method with 10 ug retroviral vectors. After 48 h, the viral supernatants were harvested and filtered. The cells were incubated overnight with viral supernatant supplemented with 8 μg/mL polybrene. Subsequently, G418 (# 077-06433; Fujifilm, Tokyo, Japan) (FLAG-tagged-PPARα, shRNAs) was used for selection. The sequences used for retroviral shRNA expression vectors targeting ELK1 are listed in Table S1.

#### RNA extraction, qRT-PCR, and RNA-sequencing analyses

Total RNA was isolated from tissues using TRIzol reagent (#15596018; Invitrogen), according to the manufacturer's protocols. Reverse transcription was performed using the PrimeScript RT Master Mix (# RR036A; Takara Bio, San Jose, CA, USA). Quantitative real-time PCR (qRT-PCR) was performed with SYBR Green fluorescent dye using a 7500 Fast PCR Machine (Applied Biosystems, Foster City, CA, USA) and TB Green Premix Ex Taq (# RR420A; Takara Bio) using the CFX384 Real-time PCR Detection System (Bio-Rad Laboratories, Hercules, CA, USA). TATA-binding protein (Tbp) served as an internal control. Primer sequences are shown in Table S1. Fold changes were calculated using the 2^–ΔΔCt^ (DDCT) method, with Tbp mRNA as a normalization control. RNA-sequencing libraries were constructed from total RNA using the SureSelect strand-specific RNA library prep system (Agilent Technologies, Santa Clara, CA, USA), according to the manufacturer’s instructions (*in vitro*, Figure 1) and using TruSeq Stranded mRNA Library Prep Kit (Illumina, San Diego, CA, USA) according to the manufacturer’s instructions (*in vivo*, Figure 1; *in vitro*, Figure 4). Library quality was determined using a bioanalyzer (Agilent Technologies). High-throughput sequencing was performed using a HiSeq 2500 instrument (Illumina) at the core facility of Hiroshima University. Raw reads for each library were mapped using TopHat v.2.0.14 against the mouse (mm10) genome. The mapped reads were converted to fragments per kilobase of exon per million fragments mapped (FPKM) using Cufflinks v.2.2.1 to determine gene expression. The MDS plot was constructed using edgeR with significant differentially expressed genes (DEGs; p < 0.05, log2 fold change > 0) upon treatment with rosiglitazone, pemafibrate, and GW9578 compared to the vehicle. Biological pathway analysis was performed using Metascape.^51^ A *de novo* motif search was performed using Homer to predict the potential transcription factors responsible for promoter regulation.

#### Oxygen consumption assays

The oxygen consumption rate was measured using an MT200A cell respirometer (Strathkelvin Instruments, Motherwell, UK), as previously described.^56^ Briefly, differentiated beige adipocytes treated with DMSO or 10 μM pemafibrate for 96 h were trypsinized and incubated with a serum-free medium. Uncoupled and non-mitochondrial cellular respiration was measured using 1 μM oligomycin and 1 μM antimycin A. To determine basal *ex vivo* oxygen consumption rates, fresh inguinal adipose tissues were extracted from mice and immediately placed into warm (37°C) PBS. Tissues were weighed (650-1,800 mg), washed in a filtered respiration buffer (PBS, 0.02% fatty acid-free bovine serum albumin, 25 mM glucose, 0.01% (vol/vol) of 100 mM Na pyruvate [Sigma]), minced with scissors, re-suspended in the respiration buffer, and placed into an MT200A cell respirometer. The obtained measurements were normalized to tissue weight.^57^

#### Chromatin immunoprecipitation assay

Differentiated adipocytes treated with a vehicle or 10 μM pemafibrate were collected, washed with cold PBS, fixed in 1% formaldehyde for 10 min at room temperature, and quenched with 125 mM glycine for 5 min at room temperature. Samples were then washed five times with cold PBS and placed in a lysis buffer containing 50 mM Tris, 1% SDS, 10 mM EDTA, and protease inhibitors, and sonicated until a mean chromatin size of 200–500 bp was achieved. Chromatin was spun at 15,000 × *g* for 10 min at 4°C, and a solution containing 16.7 mM Tris (pH 8), 1 mM EDTA, 0.01% SDS, 1.1% Triton X-100, 167 mM M NaCl, 1.2 mM EDTA, and antibodies was added, followed by overnight incubation at 4°C on a rotating platform. Dynabeads protein G (#10004D; Invitrogen) was added to samples for 2 h at 4°C on a rotating platform. Samples were washed five times with 0.1% SDS, 1% Triton X-100, 150 mM NaCl, 2 mM EDTA, and 20 mM Tris (pH 8) and twice with 0.1% SDS, 1% Triton X-100, 500 mM NaCl, 2 mM EDTA, and 20 mM Tris (pH 8). Samples were eluted overnight at 65°C in a solution containing 10 mM Tris (pH 8), 5 mM EDTA, 1% SDS, and 300 mM NaCl. The samples were subsequently treated with RNase and proteinase K and column-purified (#28004; Qiagen, Hilden, Germany). Target enrichment was calculated as a percentage of the input. Primer sequences are listed in Table S1. The commercial antibodies used were H3K27-Ac (#81735; Cell Signaling Technology, Danvers, MA, USA), ELK1 (#ab32106; Abcam), and PPARα (#GTX101098; Genetex, Irvine, CA, USA).

#### Protein interaction analysis

To confirm the interaction among PPARα, ELK1, and MED23 in beige adipocytes, nuclear protein extracts were harvested from differentiated beige adipocytes. Immunoprecipitation using anti-Sur2 (BD Pharmingen# 550429) was carried out as previously described.^58^^,^^59^ The nuclear extracts were prepared in a buffer containing 50 mM Tris (pH 8.0), 10% glycerol, 150 mM NaCl, 2 mM EDTA, 1% triton-100, and protease inhibitors. Briefly, the nuclear extracts were incubated overnight at 4°C with anti-Sur2 (#550429; BD Pharmingen) or mouse IgG (#5415; Cell signaling), washed, and eluted. The eluted materials were analyzed using western blotting to detect endogenous ELK1, PPARα, and MED23 using antibodies against ELK１(#ab32106; Abcam), PPARα (#GTX101098; GeneTex), and DRIP130 (#ab200351; Abcam). Proteins were separated in 7.5% gels (#4561025; Bio-Rad) and transferred onto PVDF membranes. ELK1, PPARα, and MED23 (DRIP130) in immunoprecipitation complex blots were detected using Veriblot for IP Detection reagent (#ab131366; Abcam).

#### Immunoblotting

The differentiated adipocytes were lysed in FLAG buffer containing 50 mM Tris (pH 8.0), 10% glycerol, 150 mM NaCl, 2 mM EDTA, 1% triton-100, and protease inhibitors (#78442; Thermo Fisher Scientific, Waltham, MA, USA). Protein concentration was measured using a Pierce BCA Protein assay kit (Thermo Fisher Scientific). Total protein lysates were boiled with 4×Laemmli Sample buffer (#161-0747; Bio-Rad Laboratories) containing 10% β-mercaptoethanol, loaded on 7.5% or 4–20% SDS-PAGE, and subsequently transferred onto PVDF membranes. The PVDF membrane blots were blocked with 5% skim milk in PBS with Tween 20 (PBS-T) and incubated overnight with rabbit anti-ELK1 (#ab32106; Abcam), anti-UCP1 (#ab10983; Abcam), anti-Total OXPHOS Rodent WB cocktail (#ab110413; Abcam) anti-PPARα (#GTX101098; GeneTex), mouse anti-α-Tubulin (#2144; Cell Signaling), or mouse anti-β-Actin (#4970; Cell Signaling). Anti-rabbit IgG (#NA934V; GE Healthcare, Chicago, IL, USA) and anti-mouse IgG (#NA931V; GE Healthcare) were used as secondary antibodies.

### Quantification and statistical analysis

The data were analyzed using Prism 9 (GraphPad Software Inc.). All data were tested for normality. Two-tailed unpaired Student’s t-tests were performed for comparisons between two groups. For multiple-group comparisons, either one-way or two-way ANOVA (or mixed-effects analysis) with Tukey’s multiple comparisons test was performed. The Mann–Whitney U test (for two-group comparisons) or Dunn's multiple comparisons test (for multiple-group comparisons) was used when samples were non-normal distributed. Correlations were assessed using Pearson’s correlation coefficient. Statistical significance was set at p < 0.05. ∗ Indicates p value < 0.05, ∗∗ indicates p value < 0.01, ∗∗∗ indicates p value < 0.001. (Figures 3E and 3F); ∗ indicates p value < 0.05, ∗∗∗ indicates p value < 0.001(Control vs. Pema), ^††^indicates p value < 0.01, ^†††^indicates p value < 0.001(Off vs. Pema), ^#^indicates p value < 0.05. Graphs were prepared using GraphPad Prism v.9. Motif enrichment was statistically analyzed using the cumulative binomial distribution (HOMER, findMotifs.pl).

## Data Availability

• High-throughput sequencing data have been deposited in the DDBJ BioProject database (https://www.ddbj.nig.ac.jp/dra/index-e.html). Sequencing data are publicly available as of the date of publication. • Accession numbers are DRA014834, DRA014833, and DRA014840. • All other data supporting the findings of this work are available from the lead contact upon reasonable request.

## Supplemental information

Document S1. Figures S1–S4

Table S1. Primer sequences used in the study
