# Supplementary material for: Selective activation of PPARα maintains thermogenic capacity of beige adipocytes
Source: iScience. 2023 Jun 19;26(7):107143. doi: 10.1016/j.isci.2023.107143 (PMC10338232; doi:10.1016/j.isci.2023.107143)
Supplement: Document S1. Figures S1–S4 [file mmc1.pdf]

iScience, Volume *26*

Supplemental information

Selective activation of PPARα maintains thermogenic capacity of beige adipocytes

Gentaro Egusa, Haruya Ohno, Gaku Nagano, Junji Sagawa, Hiroko Shinjo, Yutaro Yamamoto, Natsumi Himeno, Yoshimi Morita, Akinori Kanai, Ryuta Baba, Kazuhiro Kobuke, Kenji Oki, Masayasu Yoneda, and Noboru Hattori

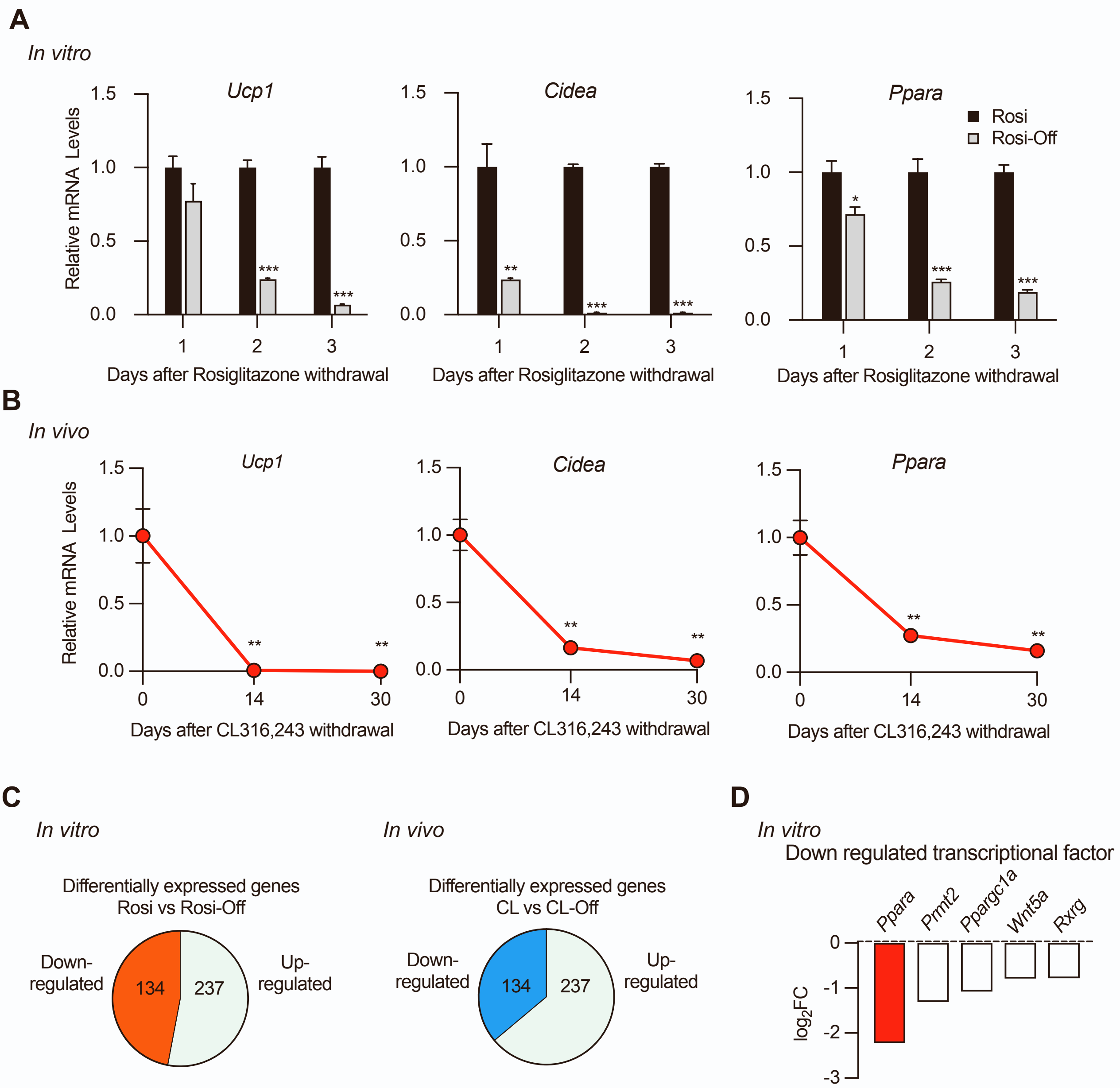

Figure S1. Deprivation of pharmacological stimuli causes beige adipocytes to lose thermogenic characteristics (Related to Figure 1). (A) Relative mRNA levels of thermogenic genes *in vitro*, with or without rosiglitazone (n = 4; mean ± SEM). (B) Relative mRNA levels of thermogenic genes *in vivo* after injection with CL316,243 for 10 days vs. withdrawal of CL after 14 and 30 days (n = 3; mean ± SEM). (C) Pie chart depicting the ratio of differentially expressed upregulated and downregulated genes obtained from RNA-seq. (D) Most downregulated transcription factors were selected using the GO term: "DNA-binding transcription factor activity" after the withdrawal of rosiglitazone *in vitro* from RNA-seq data.

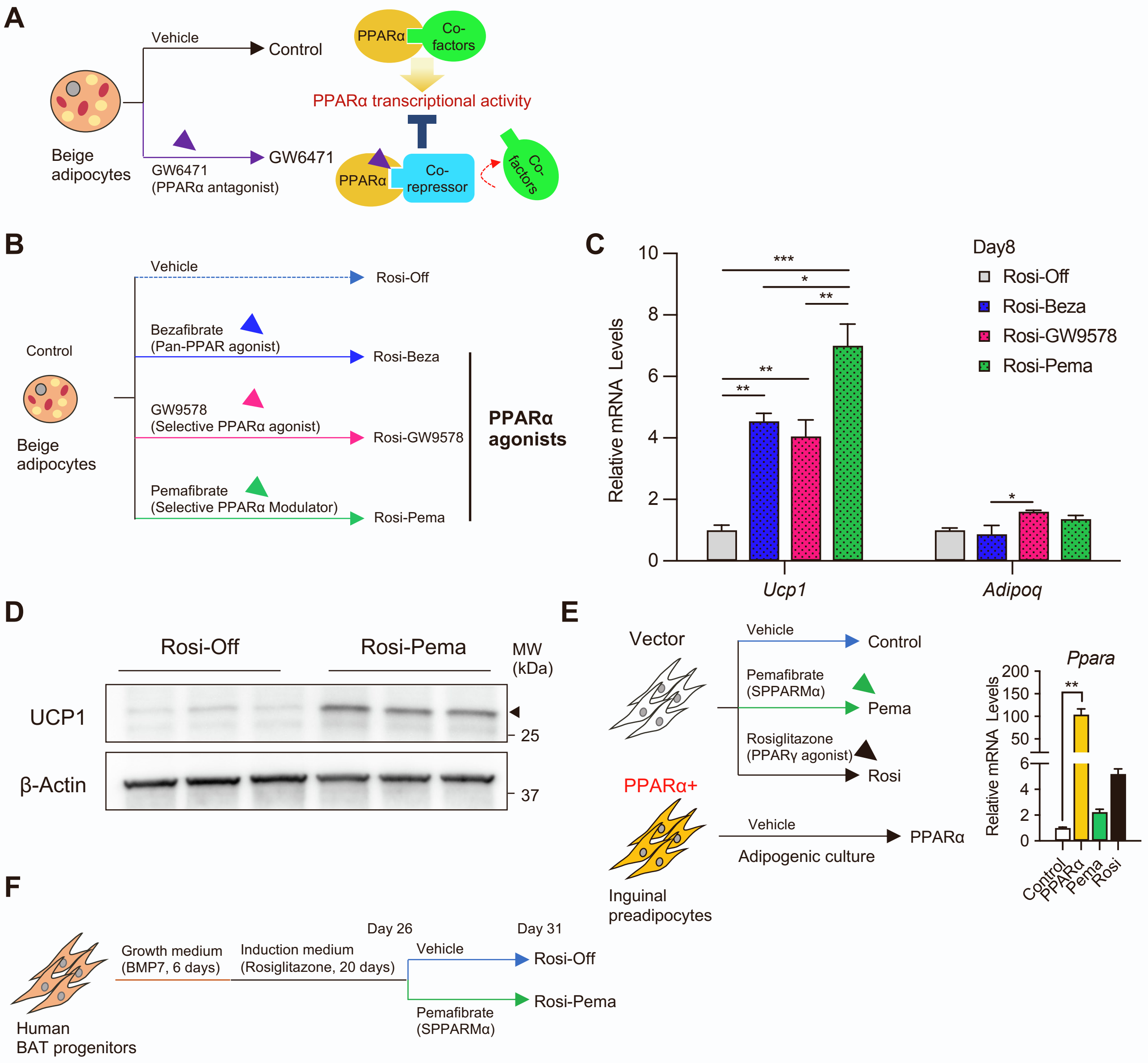

Figure S2. Effects of PPARα agonists on maintenance of thermogenic gene expressions (Related to Figure 2). (A) Schematic illustration of experiments and model for repression of PPARα transcriptional activity by GW6471. Preadipocytes were differentiated sufficiently with rosiglitazone for 5 days and vehicle or GW6471(5 μM) was added for 4 days. (B) Schematic illustration of experiments. Beige adipocytes were differentiated using rosiglitazone treatment (1 μM) for 5 days, rosiglitazone was switched to vehicle (Rosi-Off), bezafibrate (Rosi-Beza, 500 μM), GW9578 (Rosi-GW9578, 1 μM) or pemafibrate (Rosi-Pema, 10 μM) and incubated for additional 3 days. (C) Relative mRNA levels of *Ucp1* and *Adipoq* vs. those of genes in the Rosi-Off. (n = 3. mean ± SEM). (D) Western blot analysis of UCP1 protein levels in Rosi-off and Rosi-Pema on day 9 switched to vehicle or pemafibrate for 3 days. β-Actin was used as a loading control. (E) (left panel) Schematic illustration of experiments. Preadipocytes were treated with an adipogenic cocktail with vehicle (Control), pemafibrate (Pema, 10 μM), or rosiglitazone (Rosi, 1 μM). PPARα overexpressing preadipocytes were treated with an adipogenic cocktail with vehicle (PPARα). (right panel) Relative mRNA levels of Ppara in each group on day 6 (n = 4; mean ± SEM). (F) Schematic illustration of experiments. hTERT A41hBAT-SVF were pre-treated by BMP7 for 6 days. Then, they were treated with induction medium containing rosiglitazone for 20 days (Day 26, Control). Rosiglitazone were switched to vehicle (Rosi-Off) or Pemafibrate (Rosi-Pema) for additional 6 days.

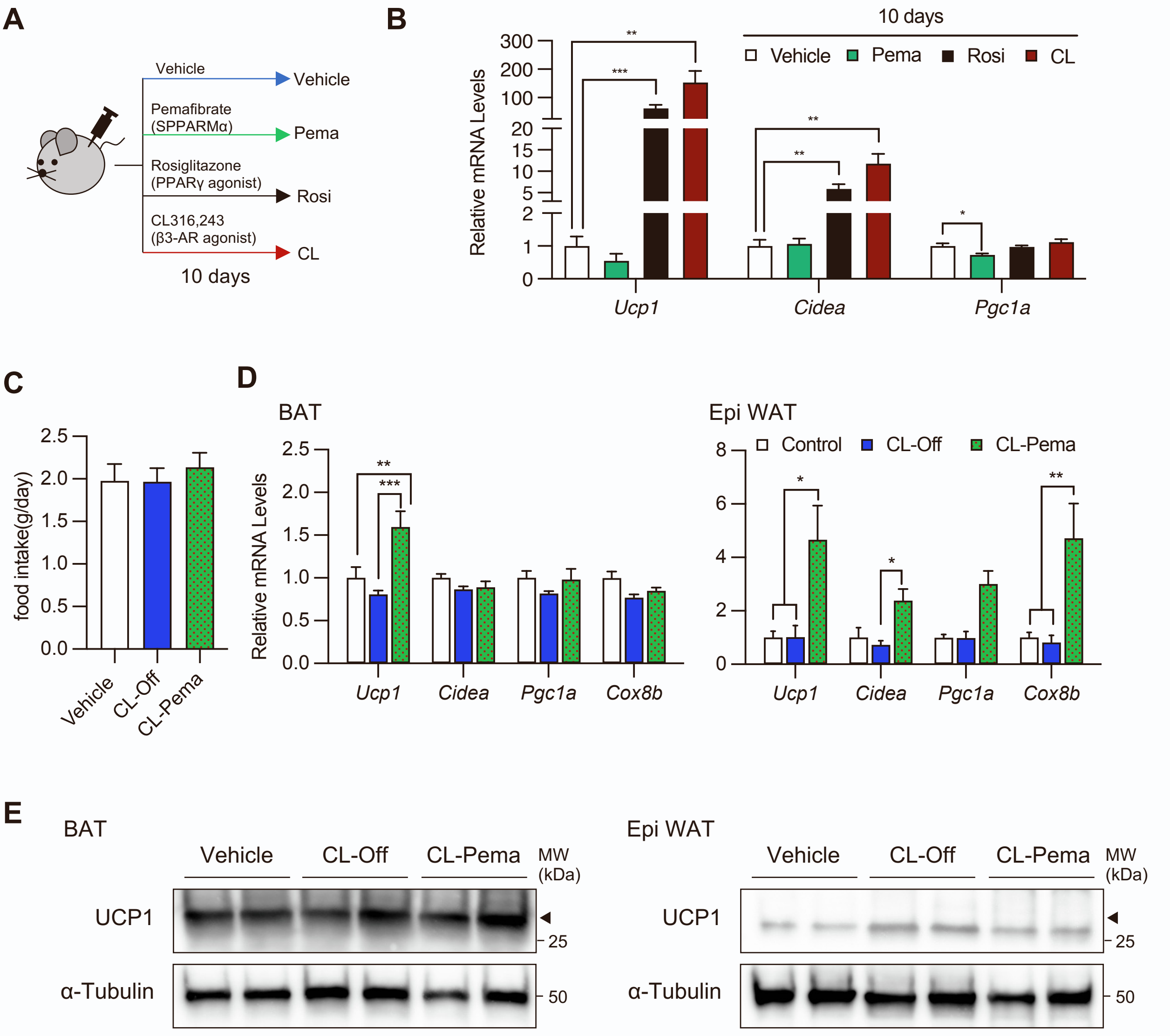

Figure S3. Effect of pemafibrate in WT or beige indeced mice model (Related to Figure 3). (A) Schematic illustration of experiments. WT B6/J male mice were treated with Vehicle, Rosiglitazone(10 mg kg-1), Pemafibrate (1 mg kg-1) or CL316,243 (1 mg kg-1) for 10 days with normal chow diet. (B) Relative mRNA levels of thermogenic genes in iWAT (n = 4–5 per group; mean ± SEM). (C-E) related to Figure 3A. (C) Food intake was measured for each group of mice every day. (D) Relative mRNA levels of thermogenic genes in iBAT and eWAT (iBAT: n = 8–10 per group; eWAT: n = 4–7 per group; mean ± SEM). (E) Western blot analysis of UCP1 protein levels in iBAT and eWAT. β-Actin was used as a loading control.

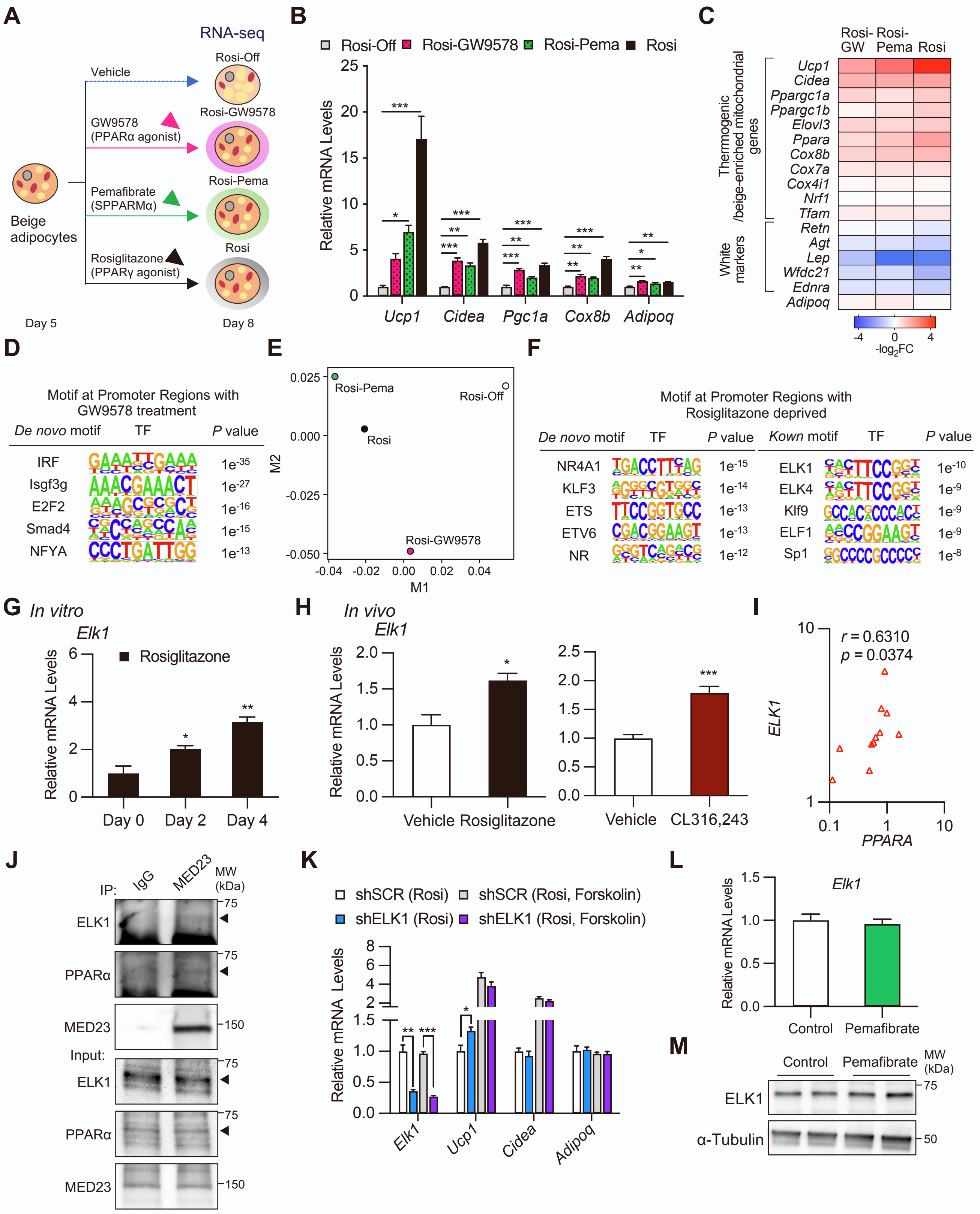
A
RNA-seq
Rosi-Off
Vehicle
Rosi-GW9578
GW9578
(PPARα agonist)
Rosi-Pema
Pemafibrate
(SPPARMα)
Rosi
Rosiglitazone
(PPARγ agonist)
Beige
adipocytes
Day 5
Day 8
B
Rosi-Off
Rosi-GW9578
Rosi-Pema
Rosi
Relative mRNA Levels
Ucp1
Cidea
Pgc1a
Cox8b
Adipoq
C
Rosi-GW
Rosi-Pema
Rosi
Thermogenic /beige-enriched mitochondrial genes
Ucp1
Cidea
Ppargc1a
Ppargc1b
Elovl3
Ppara
Cox8b
Cox7a
Cox4i1
Nrf1
Tfam
White markers
Retn
Agt
Lep
Wfdc21
Ednra
Adipoq
-log2FC
D
Motif at Promoter Regions with
GW9578 treatment
De novo motif
TF
P value
IRF
1e-35
Isgf3g
1e-27
E2F2
1e-16
Smad4
1e-15
NFYA
1e-13
E
Rosi-Pema
Rosi-Off
Rosi
Rosi-GW9578
M1
M2
F
Motif at Promoter Regions with
Rosiglitazone deprived
De novo motif
TF
P value
Kown motif
NR4A1
1e-15
KLF3
1e-14
ETS
1e-13
ETV6
1e-13
NR
1e-12
ELK1
1e-10
ELK4
1e-9
Klf9
1e-9
ELF1
1e-9
Sp1
1e-8
G
In vitro
Elk1
Rosiglitazone
Relative mRNA Levels
Day 0
Day 2
Day 4
H
In vivo
Elk1
Relative mRNA Levels
Vehicle
Rosiglitazone
Vehicle
CL316,243
I
r = 0.6310
p = 0.0374
ELK1
PPARA
J
IP:
IgG
MED23
MW
(kDa)
ELK1
PPARα
MED23
Input:
ELK1
PPARα
MED23
K
shSCR (Rosi)
shSCR (Rosi, Forskolin)
shELK1 (Rosi)
shELK1 (Rosi, Forskolin)
Relative mRNA Levels
Elk1
Ucp1
Cidea
Adipoq
L
Elk1
Relative mRNA Levels
Control
Pemafibrate
M
Control
Pemafibrate
MW
(kDa)
ELK1
α-Tubulin

Figure S4. The difference of transcriptome profiles after PPAR agonist treatment and characteristics of ELK1 in beige adipocytes (Related to Figure 4). (A) Schematic illustration of experiments. Beige adipocytes were differentiated with rosiglitazone (1 μM) treatment and followed by vehicle (control), GW9578 (1 μM, GW9578), pemafibrate (10 μM, Pema), or rosiglitazone (1μM, Rosi). (B) Relative mRNA levels of thermogenic genes in differentiated adipocytes vs. control. (n = 3; mean ± SEM). (C) Expression heatmap of thermogenic and white selective genes in the indicated treatment group vs. control. (D) *De novo* motif analysis of regions with upregulated gene treatment switched to GW9578 vs. control. (E) MDS analysis of differentially expressed genes from RNA-seq data of each group. (F) *De novo* motif analysis of regions with decreased genes after the withdrawal of rosiglitazone (see Figure 1). (G) Relative mRNA levels of *ELK1* in the differentiation of beige adipocytes treated with rosiglitazone (1 μM) *in vitro* (n = 4; mean ± SEM). (H) Relative mRNA levels of *ELK1* in differentiation of beige adipocytes treated with rosiglitazone (10 mg kg^{-1}) and CL316,243 (1 mg kg^{-1}) *in vivo* (rosiglitazone: n = 4; CL316,243: n = 4–5; mean ± SEM.) (I) Correlation of levels of ELK1 and PPARA expression (see Figure 1F). (J) Endogenous co-immunoprecipitation in differentiated beige adipocytes. Mouse IgG was used as a negative control. (K) Thermogenic genes in differentiated adipocytes treated with rosiglitazone (1 μM) ± forskolin (10 μM) expressing the indicated constructs (n = 4; mean ± SEM). (L) Relative mRNA levels of *Elk1* in the differentiation of adipocytes treated with pemafibrate (10 μM) at day 6 *in vitro* (n = 4; mean ± SEM). (M) Western blot analysis of ELK1 protein levels at day 6. α-Tubulin was used as a loading control.
